# How is the concept of charisma used in the academic literature about biodiversity conservation? A systematic map protocol

**DOI:** 10.1186/s13750-024-00353-2

**Published:** 2024-12-04

**Authors:** Maxim Isaac, Caroline S. Fukushima, Biljana Macura, Enrico Di Minin, Ricardo A. Correia

**Affiliations:** 1 Biodiversity Unit, University of Turku, Turku, 20014 Finland; 2 Helsinki Lab of Interdisciplinary Conservation Science (HELICS), Department of Geosciences and Geography, University of Helsinki, Helsinki, 00014 Finland; 3 Finnish Museum of Natural History (Luomus), University of Helsinki, Helsinki, Finland; 4 Stockholm Environment Institute (HQ), Box 24218, Stockholm, 10451 Sweden; 5 Helsinki Institute of Sustainability Science (HELSUS), University of Helsinki, Helsinki, 00014 Finland; 6 Centre for Sustainability Transitions, Stellenbosch University of Stellenbosch, Stellenbosch, South Africa

**Keywords:** Academic practice, Human preferences, Research trends

## Abstract

**Background:**

The extinction of species is a multifaceted phenomenon shaped by the complex interplay between biological and socio-cultural factors. Public and academic preferences for different species often play a direct or indirect role in influencing the conservation outlook of these species. The “charisma” of species and other components of biodiversity is often mentioned as an important factor in shaping human preferences, determining both the scope of scientific studies and justifications for such scope. Here, we present a protocol for systematically mapping the use of the concept of “charisma” in relation to biodiversity peer-reviewed academic literature focused on biodiversity conservation.

**Methods:**

The search targeting academic peer-reviewed research articles and reviews will be conducted in three publication databases, The Lens, Scopus and Web of Science (Core Collection and SciELO), and will be supplemented by search engine results from Google Scholar. Broad-scope searches will be performed in 3 different languages (English, Portuguese, and Spanish) and article screening will be performed at two stages to ensure the relevance of each entry and consistency amongst reviewers in their use of the defined inclusion criteria. The resulting systematic map of the literature will be summarised by employing a narrative synthesis approach, and through descriptive statistics and analysis of temporal trends.

**Supplementary Information:**

The online version contains supplementary material available at 10.1186/s13750-024-00353-2.

## Background

Preventing species extinctions driven by unsustainable human behaviours is amongst the greatest challenges of our time given the ongoing mass extinction event [1]. Despite growing awareness of the plight of biodiversity [2] and some successes in preventing species extinctions [3], the alarming trend of biodiversity loss persists and even shows some signs of acceleration [4] and growing complexity due to interactions with concurrent crises such as climate change, invasive species and plastic pollution [5]. Biodiversity conservation is both a moral imperative and essential to ensuring livable conditions for future generations by preserving essential ecosystem services and safeguarding the cultural underpinning of societies and communities worldwide [6].

Widespread public support is essential to address species extinctions because conservation efforts depend on human decisions, including the allocation of economic resources, and thus require behavioural changes to succeed [7]. Indeed, species extinctions can be conceptualised as a biocultural process, where biological and socio-cultural dynamics interact to define the fate of a species [8, 9]. Both public and academic attitudes and preferences towards species can have direct and indirect impacts on their survival [10]. While historical emphasis has been placed on the biological aspects of extinction, there is a growing recognition that understanding the human dimensions of conservation is crucial for effecting the transformative change needed to address species extinctions [4].

Scientists play a fundamental role in the biocultural dynamics of species extinctions through their efforts in understanding, documenting and communicating extinction processes [9]. Robust knowledge about species and their extinction processes is essential to support informed decision-making and to rally resources for conservation. However, biodiversity knowledge remains spatially and temporally uneven [11] and subject to taxonomic biases, where certain species are widely studied while others lack foundational knowledge essential for their conservation [10, 12]. Such biases are determined by a broad range of factors, including funding and resource availability for research, ease of study, and scientist’s preferences for particular taxa [13–15]. Characteristics such as large bodies, exquisite features, distinctive behaviours, striking colours, and pleasant sounds or fragrances are thought to be broadly appealing to humans and influence our perception of species [16–19]. Indeed, some species hold remarkable combinations of such traits to the point they are considered as “charismatic” [20, 21].

The term “charisma” was originally borrowed from the Latin ecclesiastical vocabulary, secularised, and has been widely applied in the context of biodiversity conservation [22]. The role of charisma in biodiversity conservation has been widely discussed in the academic literature and is thought to play a role in influencing conservation decisions, with charismatic species receiving greater volume of research attention and specificity in their conservation planning [19–21, 23]. These biases exist not only in the research attention the species receive, but seem to translate into funding gained for their conservation [24]. Furthermore, public biases in favour of charismatic species means they are often more willing to support conservation actions focusing on such species [23, 25, 26]. The adoption of the term “charisma” in the peer-reviewed academic literature related to biodiversity conservation suggests it is moving beyond a mere descriptor to become a term of perceived substance used to propose, justify and sometimes criticise targets of conservation. However, our understanding of how frequently and consistently the term is used and characterised in the academic literature remains limited.

In this protocol, we describe the approach to be used in a study that aims to assess the use of the term “charisma” in reference to biodiversity in the academic literature dealing with biodiversity conservation. Specifically, we aim to conduct a systematic map to assess how the term “charisma” is defined and to what elements of biodiversity it is commonly applied to, while incorporating studies from different research fields. We will use the results of this systematic map to describe trends in the use of the term over time and across elements of biodiversity, and explore how consistently the concept of “charisma” is defined in the academic literature. By doing so, we hope to support researchers in more clearly communicating and leveraging the concept of “charisma” in the academic literature.

## Stakeholder engagement

The topic of this systematic map was initially formulated by the lead and senior researchers. To enhance the potential relevance of the work to the conservation community, a selection of academic and non-academic stakeholders were selected from the authors’ networks to encompass viewpoints from a variety of backgrounds. We aimed to select experts with varied backgrounds and experiences in conservation, including academia, NGOs, zoos and other backgrounds.The stakeholders come from and have worked in several countries, thus offering also a good geographical coverage and contextual knowledge, and their work contains elements of socio-ecological research. They were invited via email to contribute to the scope and design of the project.

Stakeholders were invited to provide critical feedback on the original study protocol, including the research questions, search strategy, and reference inclusion/exclusion criteria, for the consideration of the review team. Suggestions considered relevant were already considered in the protocol by the authors. Stakeholders will not contribute to data collection, coding, nor to building and analysing the map database. However, stakeholders who provided feedback on the study protocol will later be invited to contribute and provide further critical feedback on the resulting synthesis, and to the manuscript reporting on the effort for consideration by the review team. In line with the authorship criteria defined by the journal (https://www.biomedcentral.com/getpublished/editorial-policies#authorship*)*, stakeholders that contribute with constructive and substantial feedback to all stages of the project mentioned above, including the protocol development and manuscript drafting, will be invited to co-author the final manuscript.

## Objective of the review

The main goal of this study is to produce a systematic map of the peer-reviewed academic literature exploring the following primary question:

“How is the concept of charisma used in the academic literature about biodiversity conservation?”.

Related to this question, we also aim to explore the following secondary questions:

1. Has the frequency of use of the term charisma in biodiversity conservation academic literature changed over time?
2. How is charisma defined in academic literature?
3. To what units of biodiversity (e.g., individuals, species, ecosystems) has the concept of charisma been applied to?

We will use the following question components:

### Population

biodiversity conservation research.

### Phenomena of interest

Use of the concept of charisma in reference to biodiversity (see 4.2.1 and Supplementary material 3).

### Context

Published peer-reviewed scientific literature in English, Spanish and Portuguese.

## Methods

This protocol complies with the Collaboration for Environmental Evidence guidelines and standards [27], and conforms with the Reporting Standards for Systematic Evidence Syntheses (ROSES) [28].

### Searching for articles

Since the goal of our systematic map is to capture the use and application of the term charisma and its variations in the academic discourse on conservation, our search strategy will include only peer-reviewed academic literature. These publications, grounded in empirical evidence and a degree of scholarly consensus emerging from the peer-review process, stand as a repository of knowledge where researchers often rigorously define, refine and debate terminologies. The regular application of a term in peer-reviewed publications therefore serves as a strong indication that it is widely accepted by the academic community. This peer reviewed literature is also used to shape theoretical frameworks in conservation and to guide research agendas. This literature ultimately informs policy development and implementation, further influencing use of specific concepts and terms across the field.

The term “charisma” is mentioned in academic literature, sometimes used interchangeably or alongside related terms such as “popular”, “iconic”, “attractive” or “flagship”. Here, we are interested specifically in the use and definition of only the term “charisma” so we have decided to restrict the searches to this term and its derivatives (e.g. charismatic). We considered expanding the scope of the work to include a broader range of related concepts and compare their use and definitions, but the volume of search results and the resources needed to screen all the terms are beyond those available in the scope of this project. We recognise that mapping other terms would be relevant to clarify the terminology in the field and propose that this protocol could serve as a template for such work in the future. Additionally, we will not restrict the subject area of the publications but we will record this data for each publication, thus allowing us to compare potential differences in the way charisma is used across different research areas.

#### Search languages

We will search the academic literature published in English, Portuguese, and Spanish languages to ensure a comprehensive and diverse coverage of relevant studies. There is increasing recognition that the scientific literature related to biodiversity conservation expands beyond that published in English and that such literature should be considered in efforts to summarise relevant information [29, 30]. The choice of including Portuguese and Spanish is rooted both in the fact that our team has native speakers of Portuguese and Spanish, and in the status of Portuguese and Spanish as two of the most widely spoken languages globally [31]. By incorporating these languages, we aim to capture a broader spectrum of studies, enhancing the inclusivity and richness of our systematic mapping exercise.

#### Search strings

As we aim to maximise the sensitivity of the searches, and encompass the various nuances and applications of the term “charisma” and its variations (e.g. “charismatic”), we will opt for using a single, broad search string for each of the languages. English searches will use the string “charism*”, and those conducted in Portuguese and Spanish will use the string “carism*” (see Table 1). Because the academic literature relevant to biodiversity conservation spans across the natural and social sciences, we will include no further filters or refining terms to the search string, thus aiming to capture all the relevant literature that matches our screening criteria (see Sect. 4.2).

**Table 1:** Summary information of the search strategy to be implemented across publication databases, including search platform, search fields, search languages, and search strings

Publication database	Search fields	Search language	Search string
Web of Science Core Collection	Article title OR Abstract OR Keywords	English	charism*
	Article title OR Abstract OR Keywords	Spanish	carism*
	Article title OR Abstract OR Keywords	Portuguese	carism*
Web of science SciELO	Topic	English	charism*
	Topic	Spanish	carism*
	Topic	Portuguese	carism*
Scopus	Article title, Abstract, Keywords	English	charism*
	Article title, Abstract, Keywords	Spanish	carism*
	Article title, Abstract, Keywords	Portuguese	carism*
The Lens	Article title OR Abstract OR Keywords	English	charism*
	Article title OR Abstract OR Keywords	Spanish	carism*
	Article title OR Abstract OR Keywords	Portuguese	carism*

#### Benchmark publications

We defined a list of benchmark publications to assess the comprehensiveness of our search (see Supplementary material 1). These benchmark papers were primarily selected manually based on our knowledge of the field. The list contains articles written in English (5 articles), Spanish (3 articles), and Portuguese (3 articles) to assess the reliability of searches in each language. We chose papers that include mentions of charisma in the context of biodiversity conservation, and that addressed the topic broadly rather than concentrating on a specific group or taxon, with the aim of representing a variety of research contexts, backgrounds and languages. We used the list of benchmark publications to validate the comprehensiveness of the searches. All the papers in the benchmark list were retrieved with our final search string.

#### Search platforms

We selected three publication databases that index academic literature for implementing the searches described above. Searches will be carried out on The Lens, Scopus and Web of Science (Core Collection and SciELO) using access provided by the University of Turku and focusing on identifying relevant academic peer-reviewed research articles and reviews. The searches will be conducted on title, abstract and keywords on all platforms to provide consistency as not all databases used offer full-text searching (see Table 1). Non-academic sources like websites and blogs as well as grey literature will not be included because we aim to focus on the peer-reviewed academic literature.

To supplement the database searches, we will also carry out more targeted searches using the Google Scholar search engine (https://scholar.google.com/). Google Scholar has proven a valuable supplementary source to publication database based maps and reviews [32, 33]. Due to the fact Google Scholar limits the results accessible from each search to 1000 publications, we will use a more targeted search string than those used in publication databases by adding the term ‘conservation’ and its equivalents in Spanish and Portuguese with the aim of increasing the relevance of results to biological conservation (see Table 1). We will retrieve all the resulting entries of these searches up to 1000 (due to the limitations imposed by the platforms) for further screening using Publish or Perish software [34].

All inaccessible records will be reported in the final map The search results will be downloaded from each source, imported into, and deduplicated in CADIMA, a free web tool for assisting throughout the systematic mapping process (https://www.cadima.info/*).*

### Article screening and study eligibility criteria

#### Article screening

Retrieved publications will be screened for relevance to our review based on two eligibility criteria. Eligible articles will be identified and retained according to the following criteria:

1. Use of a term related to the concept of “charisma” and its variations (e.g., charismatic) in the title, abstract or keywords in reference to any unit of biodiversity. Studies may encompass any element of biodiversity from individuals, through species, to landscapes, but excluding humans (see Supplementary material 2 for more detail).
2. The study is of purported relevance to biodiversity conservation, evidenced through discussion of, for example, biodiversity threats, threat status, or conservation policy, action and management. Because of the interdisciplinary nature of the conservation literature, studies will not be constrained by subject area, publication year or geography.

#### Study eligibility criteria

Eligibility will be assessed in two stages based on the criteria described above. An initial screening will be performed at the title, abstract and keyword level to ensure clearly irrelevant entries are excluded. Eligibility for some articles may not be able to be properly scrutinised based on title, abstract and keywords due to insufficient information to assess the eligibility criteria. For these articles, a subsequent screening for relevance will be done at full-text level (see below). Two reviewers will split the task of screening the articles in Portuguese and Spanish, and all the three reviewers will receive a share of articles in English.

To ensure the consistency, a subset of 100 articles will be assessed by three reviewers in both screening stages. The consistency of screening will be assessed and the percentage of agreement on screening decisions among reviewers should be found to be a minimum of 80%. If this agreement threshold is not reached then the researchers will review the differences in their application of the inclusion/exclusion criteria and re-test against a further set of articles until an 80% agreement is obtained.

A list of excluded studies in full text with reasons for their exclusion will be available in the final report.

### Study validity assessment

We will not perform a study validity assessment due to the nature of this map which is seeking to understand use of a concept in the academic literature.

### Data coding strategy

Once the relevance of each paper has been validated based on a full-text review, in line with our research questions we will extract the relevant metadata from the retrieved papers. As we are seeking to explore the use and definition of the term “charisma”, in addition to bibliographic details, the following metadata will be coded:

1. The definition of charisma as/if provided by the authors,
2. The unit of biodiversity related to charisma, depending on whether charisma is applied to a specific individual, a species or group of species, or an ecosystem or landscape.
3. The type of the definition (e.g. Explicit definition, Example-based definition, Citation, Other or No definition).
4. Where the concept of charisma is used in the text (e.g. abstract, introduction, objectives, methods, results or discussion),

Supplementary material 2 is a detailed coding handbook.

These coding elements will be input into CADIMA our proposed reviewing software to easily collate them for analysis.

The consistency of the data coding will be examined by multiple reviewers on a subsample of 20 papers prior to conducting the full coding exercise, with disparity in extracted information reconciled through group discussion amongst authors. If additional coding categories are added during the review process, changes to the original coding will be reported in the final systematic map report.

### Study mapping and presentation

We will provide a narrative synthesis of all included publications. Descriptive statistics and temporal trends will be presented to summarise bibliographic information such as the type of journal, authorship and year of publication (see SRQ1), the types of definition used for charisma in the literature (SRQ2), classified according to whether they are an explicit definition, example-based definitions or citation-based definition (see Supplementary material 2), and the specific units of biodiversity to which the term “charisma” and its variations are applied (SRQ3), depending on whether charisma is applied to a specific individual, a species or group of species, or an ecosystem or landscape (see Supplementary material 2) Figures and tables can be used to supplement the narrative when necessary. All meta-data to be extracted is outlined in the coding book (see Supplementary material 2).

## Electronic supplementary material

Below is the link to the electronic supplementary material.

Supplementary Material 1

Supplementary Material 2

Supplementary Material 3

## Data Availability

Data sharing is not applicable to this protocol article as no datasets were generated or analysed during its development. In line with the principles of open science and transparency, the data that supports the findings of this systematic mapping will be made openly available in suitable repositories to be defined at the time of publication of the final systematic map.
