# Supplementary material for: How is the concept of charisma used in the academic literature about biodiversity conservation? A systematic map protocol
Source: Environ Evid. 2024 Dec 4;13:29. doi: 10.1186/s13750-024-00353-2 (PMC11616242; doi:10.1186/s13750-024-00353-2)
Supplement: Supplementary file 1 — Supplementary Material 1 [file 13750_2024_353_MOESM1_ESM.docx]

Benchmark papers: How is the concept of charisma used in the academic literature about biodiversity conservation? A systematic map protocol

1.

Romagnoli FC, Scabin A, Pegas F de V. BOTO-VERMELHO (Inia geofrensis): ESPÉCIE BANDEIRA PARA PROMOÇÃO DO ECOTURISMO NA AMAZÔNIA CENTRAL, BRASIL. Revista Brasileira de Ecoturismo (RBEcotur). 2011;4. doi:[10.34024/rbecotur.2011.v4.5953](https://doi.org/10.34024/rbecotur.2011.v4.5953)

2.

Krause M, Robinson K. “Charismatic Species and Beyond: How Cultural Schemas and Organisational Routines shape Conservation.” Conservation and Society. 2017;15: 313–321.

3.

Arango X, Rozzi R, Massardo F, Anderson CB, Ibarra YT. DESCUBRIMIENTO E IMPLEMENTACIÓN DEL PÁJARO CARPINTERO GIGANTE (CAMPEPHILUS MAGELLANICUS) COMO ESPECIE CARISMÁTICA: UNA APROXIMACIÓN BIOCULTURAL PARA LA CONSERVACIÓN EN LA RESERVA DE BIOSFERA CABO DE HORNOS. Magallania. 2007. doi:[10.4067/S0718-22442007000200006](https://doi.org/10.4067/S0718-22442007000200006)

4.

Vergara OE, Jerez V. Estado de conservación de Chiasognathus granti Stephens 1831 (Coleóptera: Lucanidae) en Chile. Rev chil hist nat. 2009;82. doi:[10.4067/S0716-078X2009000400010](https://doi.org/10.4067/S0716-078X2009000400010)

5.

Gallina S, González-Romero A, Gallina S, González-Romero A. La conservación de mamíferos medianos en dos reservas ecológicas privadas de Veracruz, México. Revista mexicana de biodiversidad. 2018;89: 1245–1254. doi:[10.22201/ib.20078706e.2018.4.2476](https://doi.org/10.22201/ib.20078706e.2018.4.2476)

6.

Galetti M, Carmignotto AP, Percequillo AR, Santos MC de O, Ferraz KMPM de B, Lima F, et al. Mammals in São Paulo State: diversity, distribution, ecology, and conservation. Biota Neotrop. 2022;22: e20221363. doi:[10.1590/1676-0611-BN-2022-1363](https://doi.org/10.1590/1676-0611-BN-2022-1363)

7.

Bulte EH, van Kooten GC. Marginal Valuation of Charismatic Species: Implications for Conservation. Environmental and Resource Economics. 1999;14: 119–130. doi:[10.1023/A:1008309816658](https://doi.org/10.1023/A:1008309816658)

8.

Lorimer J. Nonhuman Charisma. Environ Plan D. 2007;25: 911–932. doi:[10.1068/d71j](https://doi.org/10.1068/d71j)

9.

Hausmann A, Toivonen T, Heikinheimo V, Tenkanen H, Slotow R, Di Minin E. Social media reveal that charismatic species are not the main attractor of ecotourists to sub-Saharan protected areas. Sci Rep. 2017;7: 763. doi:[10.1038/s41598-017-00858-6](https://doi.org/10.1038/s41598-017-00858-6)

10.

Jarić I, Courchamp F, Correia RA, Crowley SL, Essl F, Fischer A, et al. The role of species charisma in biological invasions. Front Ecol Environ. 2020 [cited 12 Apr 2020]. doi:[10.1002/fee.2195](https://doi.org/10.1002/fee.2195)

11.

Tortato FR, Ribas C, Concone HVB, Hoogesteijn R. Turismo de observação de mamíferos no Pantanal. bcnaturais. 2022;16: 351–370. doi:[10.46357/bcnaturais.v16i3.814](https://doi.org/10.46357/bcnaturais.v16i3.814)
