# Supplementary material for: How is the concept of charisma used in the academic literature about biodiversity conservation? A systematic map protocol
Source: Environ Evid. 2024 Dec 4;13:29. doi: 10.1186/s13750-024-00353-2 (PMC11616242; doi:10.1186/s13750-024-00353-2)
Supplement: Supplementary file 2 — Supplementary Material 2 [file 13750_2024_353_MOESM2_ESM.docx]

Coding handbook for ‘How is the concept of charisma used in the academic literature about biodiversity conservation? A systematic map protocol’

The following information will be collected for every accessible paper that has passed the screening stages for the map:

- Where is charisma represented in the paper?
  - Heading only

*Charisma term mentioned in Title, Abstract and/or Keywords only*

- - Full-text (excluding objectives)

*Charisma term mentioned also in the full-text but not in the objectives*

- - Objectives

*Charisma term mentioned in the objectives of the study*

- Journal subject area
- Year of publication
- Authorship classification
   *How is the article authored?*
  - Single author
     *Study is authored by one person. If not, classify based on one of the criteria below*
  - Authors based at the same institution
     *All authors have at least one common affiliation*
  - Authors based on the same nation
     *All authors are affiliated to institutions within the same country*
  - International group of authors
     *Authors belong to institutions from multiple countries*
- First author country of affiliation
  - *The country or countries with which the lead author is affiliated (separate with commas if multiple)*
- Type of study
- Unit of biodiversity associated with charisma
  - Individual

*Study refers to specific individuals (e.g. Cecil the Lion)*

- - Species

*Study refers to one or multiple taxa (e.g. megafauna)*

- - Landscape

*Study refers to a geographic unit (includes ecosystems, habitats, etc.; e.g., the Amazon)*

- - Other
- Unit of biodiversity as mentioned in text (copy from full-text)
- Is the unit described as charismatic the main subject of the paper
  - Yes/No
- Definition type
  - Explicit definition
     *Study provides an explicit definition of the term 'charisma'*
  - Example-based definition
     *Study provides example(s) of charismatic species*
  - Citation
     *Study provides a citation when referring to the term 'charisma'.*
  - Other
    - A definition that does not fit the above categories
  - No definition provided
    - Study does not provide defintion
- Definition text (copy from full-text)
