# Supplementary material for: How is the concept of charisma used in the academic literature about biodiversity conservation? A systematic map protocol
Source: Environ Evid. 2024 Dec 4;13:29. doi: 10.1186/s13750-024-00353-2 (PMC11616242; doi:10.1186/s13750-024-00353-2)
Supplement: Supplementary file 3 — Supplementary Material 3 [file 13750_2024_353_MOESM3_ESM.docx]

| **Item number** | **Section / sub-section** | **Topic** | **Description** | **Further explanation** | **Checklist/Meta-data** | **Author response** | **Comments** |
| --- | --- | --- | --- | --- | --- | --- | --- |
| 1 | Title | Title | The title must indicate that it is a systematic map protocol, and must indicate if it is an update/amendment: e.g. "A systematic map update protocol...". | The title should normally be the same or very similar to the review question. | Meta-data |  |  |
| 2 | Type of review | Type of review | Select one of the following types of review: systematic map, systematic map update, systematic map amendment | See CEE Guidance on systematic mapping [1], and on amendments and updates [2] | Meta-data | systematic map |  |
| 3 | Authors contacts | Authors contacts | The full names, institutional addresses, and email addresses for all authors must be provided. |  | Checklist | Yes |  |
| 4 | Abstract | Structured summary | Abstract must not exceed 350 words and must include two sections 1) Background, the context and purpose of the review, including the review question; 2) Methods, how the review will be conducted and the outputs that are expected (specifically mention search strategy, inclusion criteria, critical appraisal, data extraction and synthesis). |  | Checklist | Yes |  |
| 5 | Background | Background | Describe the rationale for the review in the context of what is already known. Protocol must indicate why this study was necessary and what it aims to contribute to the field. | A theory of change and/or conceptual model can be presented that links the intervention or exposure to the outcome. | Checklist | Yes |  |
| 6 | Stakeholder engagement | Stakeholder engagement | The planned/actual role of stakeholders throughout the review process (e.g. in the formulation of the question) must be described and explained (using a broad definition of ‘stakeholder’, including e.g. researchers, funders and other decision-makers; see [3]) |  | Checklist | Yes |  |
| 7 | Objective of the review | Objective | Describe the primary question and secondary questions (when applicable). | The primary question is the main question of the review. Secondary questions are usually linked to sources of heterogeneity (effect modifiers). | Checklist | Yes |  |
| 8 |  | Definitions of the question components | Break down and summarise question key elements e.g. population, intervention(s)/exposure(s), comparator(s), and outcome(s). | For other question types see [4,5] | Meta-data |  |  |
|  | Methods |  |  |  |  |  |  |
| 9 | Searches | Search strategy |  | Details regarding search strategy testing should be provided. | Checklist | Yes |  |
| 10 |  | Search string | Provide Boolean-style full search string and state the platform for which the string is formatted (e.g. Web of Science format) |  | Meta-data |  |  |
| 11 |  | Languages – bibliographic databases | List languages to be used in bibliographic database searches. |  | Meta-data |  |  |
| 12 |  | Languages – grey literature | List languages to be used in organizational websites searches and web-based search engines. |  | Not performed |  |  |
| 13 |  | Bibliographic databases | Provide the number of bibliographic databases to be searched. |  | Meta-data |  |  |
| 14 |  | Web – based search engines | Provide the number of web – based search engines to be searched. |  | Not performed |  |  |
| 15 |  | Organisational websites | Provide the number of organisational websites to be searched. |  | Not performed |  |  |
| 16 |  | Estimating the comprehensiveness of the search | Describe the process by which the comprehensiveness of the search strategy was assessed (i.e. list of benchmark articles). |  | Checklist | Yes | A list of benchmark papers was assembled and can be found in S2 Text: benchmark references |
| 17 |  | Search update | Describe any plans to update the searches during the conduct of the review. | Optional. A search update is good practice if original searches were performed more than two years prior to review completion. | Checklist | n/a |  |
| 18 | Article screening and study inclusion criteria | Screening strategy | Describe the methodology for screening articles/studies for relevance/eligibility. |  | Checklist | Yes | Screening was conducted at two stages, title abstract and keywords and then again at full text. it was screen according to two criteria, use of the term charisma in relation to biodiversity and relevance to conservation. (detailed in greater detail in section 3.3. of the protocol) |
| 19 |  | Consistency checking | Describe clearly the process for checking consistency of decisions including the levels at which consistency checking will be undertaken and estimated proportion of articles/studies that will be screened and checked for consistency by two or more reviewers (e.g. Titles (10%), abstracts (10%), full text (10%)). |  | Checklist | Yes | A subset of 200 articles will be assessed by three reviewers at title and abstract levels and again at full text. The consistency of screening will be assessed using Kappa tests. (for more information see 3.3.1 of protocol) |
| 20 |  | Inclusion criteria | Describe the inclusion criteria used to assess relevance of identified articles/studies. These must be broken down into the question key elements (e.g. relevant subject(s), intervention(s)/exposure(s), comparator(s), outcomes, study design(s)) and any other restrictions (e.g. date ranges or languages). |  | Checklist | Yes | This will be the same as the screening criteria as we are interested in as broad a range of pepers as possible using charisma in relation to biodiversity (see protocol section 3.3 ) |
| 21 |  | Reasons for exclusion | State that you will provide a list of articles excluded at full text with reasons for exclusion. |  | Checklist | Yes |  |
| 22 | Critical appraisal | Critical appraisal strategy | Describe here the method you propose for critical appraisal of study validity (including assessment of individual studies and the evidence base as a whole). | Optional | Checklist | n/a |  |
| 23 |  | Critical appraisal used in synthesis | Describe how the information from critical appraisal will be used in synthesis. | Optional | Checklist | n/a |  |
| 24 |  | Consistency checking | Describe how repeatability of critical appraisal of study validity will be tested. | Optional | Checklist | n/a |  |
| 25 | Data extraction | Meta-data extraction and coding strategy | Describe the method for meta-data extraction and coding for studies (potentially providing forms/data sheets (ideally piloted), list if variables to be extracted as meta-data and those that will be coded). |  | Checklist | Yes | Meta-data to be extracted is detailed in S3 Text: coding book |
| 26 | Data synthesis and presentation | Narrative synthesis strategy | Describe methods to be used for narratively synthesising the evidence base in the form of descriptive statistics, tables (including SM database) and figures. | Vote-counting (tallying of studies based on the direction or significance of their findings) must be avoided. May include a summary of the outputs of critical appraisal of the evidence base as a whole (if planned to be performed in SM). | Checklist | Yes | Intended narrative approach detailed in protocol 3.4 |
| 27 |  | Knowledge gap and cluster identification strategy | Describe the methods to be used to identify and/or prioritise key knowledge gaps (unrepresented or underrepresented subtopics that warrant further primary research) and knowledge clusters (well-represented subtopics that are amenable to full synthesis via systematic review). |  | Checklist | No |  |
| 28 |  | Demonstrating procedural independence | Describe the role of systematic reviewers (who have also authored articles to be considered within the review) in decisions regarding inclusion or critical appraisal of their own work. | Reviewers who have authored articles to be considered within the review should be prevented from unduly influencing inclusion decisions, for example by delegating tasks appropriately. | Checklist | Yes | Any articles that are identified during the review process as authored by any of the reviewers will be handled separately and the author(s) will not be involved in the final decision to include or not the manuscript in the final map. |
| 29 | Declarations | Competing interests | Describe of any financial or non-financial competing interests that the review authors may have. |  | Checklist | Yes | Detailed in protocol section 5. |
